# Reliable Contactless Monitoring of Heart Rate, Breathing Rate, and Breathing Disturbance During Sleep in Aging: Digital Health Technology Evaluation Study

**DOI:** 10.2196/53643

**Published:** 2024-08-27

**Authors:** Kiran K G Ravindran, Ciro della Monica, Giuseppe Atzori, Damion Lambert, Hana Hassanin, Victoria Revell, Derk-Jan Dijk

**Affiliations:** 1 Surrey Sleep Research Centre Guildford United Kingdom; 2 UK Dementia Research Institute, Care Research and Technology Centre at Imperial College, London, United Kingdom, and the University of Surrey, Guildford London United Kingdom; 3 Surrey Clinical Research Facility, University of Surrey Guildford United Kingdom; 4 NIHR Royal Surrey Clinical Research Facility Guildford United Kingdom

**Keywords:** Withings Sleep Analyzer, Emfit, Somnofy, contactless technologies, vital signs, evaluation, apnea-hypopnea index, wearables, nearables

## Abstract

**Background:**

Longitudinal monitoring of vital signs provides a method for identifying changes to general health in an individual, particularly in older adults. The nocturnal sleep period provides a convenient opportunity to assess vital signs. Contactless technologies that can be embedded into the bedroom environment are unintrusive and burdenless and have the potential to enable seamless monitoring of vital signs. To realize this potential, these technologies need to be evaluated against gold standard measures and in relevant populations.

**Objective:**

We aimed to evaluate the accuracy of heart rate and breathing rate measurements of 3 contactless technologies (2 undermattress trackers, Withings Sleep Analyzer [WSA] and Emfit QS [Emfit]; and a bedside radar, Somnofy) in a sleep laboratory environment and assess their potential to capture vital signs in a real-world setting.

**Methods:**

Data were collected from 35 community-dwelling older adults aged between 65 and 83 (mean 70.8, SD 4.9) years (men: n=21, 60%) during a 1-night clinical polysomnography (PSG) test in a sleep laboratory, preceded by 7 to 14 days of data collection at home. Several of the participants (20/35, 57%) had health conditions, including type 2 diabetes, hypertension, obesity, and arthritis, and 49% (17) had moderate to severe sleep apnea, while 29% (n=10) had periodic leg movement disorder. The undermattress trackers provided estimates of both heart rate and breathing rate, while the bedside radar provided only the breathing rate. The accuracy of the heart rate and breathing rate estimated by the devices was compared with PSG electrocardiogram-derived heart rate (beats per minute) and respiratory inductance plethysmography thorax-derived breathing rate (cycles per minute), respectively. We also evaluated breathing disturbance indexes of snoring and the apnea-hypopnea index, available from the WSA.

**Results:**

All 3 contactless technologies provided acceptable accuracy in estimating heart rate (mean absolute error <2.12 beats per minute and mean absolute percentage error <5%) and breathing rate (mean absolute error ≤1.6 cycles per minute and mean absolute percentage error <12%) at 1-minute resolution. All 3 contactless technologies were able to capture changes in heart rate and breathing rate across the sleep period. The WSA snoring and breathing disturbance estimates were also accurate compared with PSG estimates (WSA snore: *r*^2^=0.76; *P*<.001; WSA apnea-hypopnea index: *r*^2^=0.59; *P*<.001).

**Conclusions:**

Contactless technologies offer an unintrusive alternative to conventional wearable technologies for reliable monitoring of heart rate, breathing rate, and sleep apnea in community-dwelling older adults at scale. They enable the assessment of night-to-night variation in these vital signs, which may allow the identification of acute changes in health, and longitudinal monitoring, which may provide insight into health trajectories.

**International Registered Report Identifier (IRRID):**

RR2-10.3390/clockssleep6010010

## Introduction

### Background

Vital signs measured in clinical practice include heart rate, breathing rate, blood pressure, and body temperature. These serve as objective measurements of normal physiological functions and play a fundamental role in the assessment of health [1,2]. With aging, there is an increased incidence of functional limitations and chronic conditions, including hypertension, coronary heart disease, stroke, type 2 diabetes, and sleep apnea. More than 65% of adults aged ≥65 years report multimorbidity. The presence of uncontrolled comorbidities reduces the quality of life; leads to loss of independence; and increases the incidence of falls, hospitalization, and mortality [3-6].

Standardized, continuous vital signs monitoring systems, when implemented for at-home monitoring and care of older adults, including people living with dementia, can serve as an important tool for early identification of changes in health, improve care in older people, and reduce the burden on the health care system [7-10]. Commercially available wearable devices (wearables) and contactless technologies (nearables) are increasingly used for home monitoring and have the potential to enable remote health monitoring and promote independent living [11-18]. These technologies offer secure digital infrastructure that allows reliable and seamless transfer of collected data to cloud servers and can facilitate long-term remote monitoring opportunities for health care.

Wearables are widely used for continuous, at-home monitoring of heart rate, and some have been evaluated in-laboratory settings, predominantly in younger age groups [17-25]. Although several wearables have been shown to be acceptable for older adults, lower technology adoption rate; user comfort trade-off; and burden of maintenance (eg, removal during some daily activities such as showers, periodic recharging, and mobile app use) may make them unsuitable for long-term use in people with cognitive impairment due to their associated behavioral and psychological symptoms [23,26].

Contactless technologies can be embedded in the living environment such as under the bed mattress (undermattress devices or bed sensors) or on the bedside table (eg, bedside radars) and allow contactless monitoring when the user is in bed [27]. They are powered by the mains and securely stream the collected data wirelessly. They use several contactless sensing modalities to measure a composite signal (ballistographic signal) containing movements resulting from breathing and cardiac activity to extract vital signs (heart rate and breathing rate) information. The bedside radars use the Doppler radar technique, while the undermattress devices use several technologies, such as electromechanical films and pneumatic sensors [27,28]. Due to their inconspicuous nature and low maintenance, they do not pose any of the burdens imposed by wearables and are an ideal tool for continuous monitoring of vital signs, behavioral information, and sleep in community-dwelling older adult populations with long-term conditions and in people living with dementia [13,14,29,30].

### Objective

To realize the potential of contactless technologies for monitoring vital signs such as heart rate and breathing rate in the community, the validity of their measurements needs to be evaluated in relevant populations. While the validity of the heart rate and breathing rate estimates collected from a few contactless technologies has been evaluated in younger populations, to the best of our knowledge, there are no vital signs evaluation studies in older adults (aged >65 years) although these devices have been implemented in longitudinal studies [13,29,31-33]. Here, we evaluated the validity of heart rate and breathing rate measurements collected from 3 contactless technologies (a bedside radar and 2 undermattress devices) against polysomnography (PSG) electrocardiogram (ECG)-derived heart rate and respiratory inductance plethysmography thorax (RIP thorax)-derived breathing rate in a laboratory setting. Throughout this paper, we have used the term vital signs to denote heart rate or breathing rate and vice versa. The evaluation addresses aspects of overnight average estimates; the ability to capture overnight trends; variability in heart rate and breathing rate; and accuracy at different sleep stages and time resolutions (60-, 10-, and 1-minute intervals) of estimates. We also discuss the data collection reliability in a home environment and summary estimates of breathing disturbance from the devices. To enhance the relevance of this study, we applied liberal inclusion and exclusion criteria for the participant selection, such that several participants had comorbidities that were representative of the general older population.

## Methods

### Cohort Characteristics

The study data were collected at home for a period of 7 to 14 days, followed by an overnight laboratory session (with full PSG) at the Surrey Sleep Research Centre in 2 cohorts (cohort 1: n=18, from January to March 2020; cohort 2: n=17, from June to November 2021). The participant group consisted of 35 individuals (men: n=21, 60%) aged between 65 and 83 (mean 70.8 SD 4.9) years. The participants were identified and recruited through the Surrey Clinical Research Facility. All participants attended an in-person screening visit, during which a range of assessments were completed. These included medical history (self-reported), the Pittsburgh Sleep Quality Index, the International Consultation on Incontinence Questionnaire-Urinary Incontinence, intermediate activities of daily living questionnaire, and the Epworth Sleepiness Scale. The vital signs (heart rate, breathing rate, blood pressure, and temperature) were also collected and reviewed to determine inclusion in the study. To ensure the ecological validity of the collected data in this population, participants with stable comorbidities such as hypertension, type 2 diabetes, arthritis, and so on, were included in the study if their comorbidity and concomitant medications were stable and were not considered to pose a safety risk. Eligible participants had to be able to independently perform activities of daily life, as assessed by the intermediate activities of daily living questionnaire, and comply with study procedures. Participants were provided with detailed information about the study and provided written informed consent before any study procedures were performed. A detailed description of the inclusion and exclusion criteria can be found in our previous publications, in which we evaluated the ability of these technologies to estimate sleep timing in the home environment and sleep stages as defined by PSG [34,35].

### Ethical Considerations

The study received a favorable opinion from the University of Surrey Ethics Committee (UEC-2019-065-FHMS) and was conducted in line with the Declaration of Helsinki and the principles of Good Clinical Practice. Potential participants were given detailed information about the study protocol, and they provided written informed consent before any study procedures were performed. Complete details of the study, along with the data management, privacy, and confidentiality measures, are discussed in the protocol [36].

### Study Protocol

The undermattress devices (Withings Sleep Analyzer [WSA]; Withings] and Emfit QS; Emfit Ltd) were deployed both in the laboratory and at home, while the bedside radar (Somnofy; VitalThings) was only used in the laboratory. The participants also used an actigraphy device (Actiwatch Spectrum [AWS]; Philips Respironics) and maintained a consensus sleep diary at home [37]. During the home deployment period, the contactless technologies did not require any manual intervention or maintenance by the participants, and the data was transmitted automatically via Wi-Fi. To ensure the anonymity of the data, no personally identifiable participant information was added to the device applications, and a portable Wi-Fi router was used for data transfer. The contactless devices remained active and collected data continuously for the entire period of the study. The participants wore AWS on their nondominant hands throughout the study period, removing them briefly in scenarios that could lead to the device becoming wet. AWS data have been reported elsewhere [34].

After the home data collection period, 1 overnight full clinical PSG recording was conducted, which included an extended time in bed of 10 hours. On average, the participants slept 385.97 (SD 65.67) minutes in the sleep laboratory as measured via PSG and 405.96 (SD 84.00) minutes as measured via consensus sleep diary at home [34,35]. The WSA was used in both cohort 1 and cohort 2, while the Somnofy and Emfit were deployed only in cohort 2. The data from the contactless technologies were collected simultaneously along with PSG in the laboratory and with AWS and sleep diary at home. Empatica E4 (Empatica Srl), a wrist-worn device that collects activity and photoplethysmography, was also deployed during the laboratory session, and our evaluation of this device is reported elsewhere [26]. All devices and PSG data were collected for the entire 10-hour in-bed period in the laboratory. A detailed description of the study protocol is given in the protocol [36].

### The Reference Vital Signs Data

During the in-laboratory session, PSG data were collected using the SomnoHD system (SOMNOmedics GmbH). The collected data included electroencephalography (256 Hz; F3-M2, C3-M2, O1-M2, F4-M1, C4-M1, and O2-M1); ECG (modified lead II subclavicular electrode placement; 256 Hz); RIP thorax and abdomen (128 Hz); photoplethysmography (128 Hz); electromyography (256 Hz, both submental and limb); and electrooculography (256 Hz; E2-M1 and E1-M2). In addition, data on the snore sensor (256 Hz) and airflow via nasal cannula and flow thermistor (128 Hz) were also collected. Sleep was scored at 30-second intervals in the Domino software environment as per American Academy of Sleep Medicine (AASM) guidelines by 2 independent scorers (a registered polysomnographic technologist and a trained scorer), and a consensus hypnogram was generated [38]. The sleep hypnogram contains 5 stages: rapid eye movement (REM), stage 1 of non-REM sleep (N1), stage 2 of non-REM sleep (N2), stage 3 of non-REM sleep (N3), and wake. The apnea-hypopnea index (AHI; number of apnea/hypopnea events per hour) and period limb movement index (PLMI; number of period limb movement events per hour) were determined by the Registered Polysomnographic Technologist using scoring rules recommended by AASM. An apnea was scored when there was a ≥90% drop in airflow lasting for at least 10 seconds, while a hypopnea was scored using the 3% drop in oxygen saturation and an arousal in the electroencephalogram criteria. The severity of apnea was determined using the following thresholds as per AASM guidelines: AHI score of <5 (normal), 5 to <15 (mild apnea), 15 to <30 (moderate apnea), and ≥30 (severe). A periodic limb movement event was scored when at least 4 consecutive limb movements occurred, each separated from the preceding limb movement by at least 5 seconds but not more than 90 seconds apart. PLMI of >15 seconds is used as the cutoff for the presence of periodic limb movement disorder [39,40]. In addition, participants with cardiac arrhythmia were identified using the arrhythmia index generated by the Domino software and verified by visual inspection of the record.

The PSG data were exported as standard EDF+ files along with recording markers and the consensus hypnogram. The ECG from the PSG was used for the extraction of the heart rate reference data, while the RIP thorax was used as the breathing rate reference data. For one of the participants in whom RIP thorax was unavailable, RIP abdomen was used to create the breathing rate reference data. MATLAB 2021b was used for all data analyses reported. The RR intervals used for the computation of the heart rate were derived from the ECG using the PhysioNet cardiovascular signal toolbox and a well-evaluated beat detection toolbox [41,42]. This beat-to-beat information was used to estimate reference heart rate (beats per minute [bpm]) at 30-second intervals, which is the same as the PSG hypnogram. For extracting the breathing rate (cycles per minute [cpm]) from the RIP thorax signal at 30-second intervals, the RRest package was used [43,44].

### Contactless Technologies: Data Overview

The WSA and Somnofy data (json format) were downloaded using the respective manufacturer’s application programming interface, while the Emfit data (CSV format) were downloaded from the manufacturer’s web interface. All the compared contactless technologies (WSA, Emfit, and Somnofy) provided breathing rate data, while only the undermattress devices (WSA and Emfit) provided heart rate data.

The devices provided vital signs (heart rate and breathing rate) data and sleep hypnograms at different resolutions (WSA: 60 seconds; Emfit and Somnofy: 30 seconds). The WSA and Somnofy heart rate and breathing rate estimations were available at 60 and 30-second resolutions (same as the respective device hypnogram resolution), while the Emfit estimated heart rate at a 4-second resolution. These 4-second estimates were averaged to generate estimates at 30-second intervals to match the Emfit sleep label intervals. To allow data analysis relative to local time, daylight savings correction was applied to the Coordinated Universal Time timeseries generated by the devices. The sleep hypnograms generated by the devices contain 4 stages: deep sleep (DS=N3), light sleep (LS=N2/N1), REM, and wake.

Apart from heart rate and breathing rate, Emfit generates continuous heart rate variability and activity measures, while Somnofy provides the estimates of movement and environmental variables such as ambient light, sound, temperature, pressure, humidity, and indoor air quality, which were out of scope for this evaluation and are not discussed here.

Both at home and in the laboratory, all devices were connected to the same network, and the devices used the manufacturer’s time synchronization protocol such as network time protocol to timestamp the data. Although this ensured that the devices were synchronized to local time, we performed another synchronization step to allow an accurate comparison of the data between the devices. The device vital signs measures were aligned to the PSG reference vital signs estimates via cross-correlation between the device and PSG vital signs and hypnograms, and the lag (within a 5-minute window) that provided the best alignment of both the vital signs data and hypnograms was then applied. The WSA data were converted from 60- to 30-second intervals by upsampling. Epochs in the PSG and device hypnograms that were scored as artifacts or no presence were excluded from the assessment.

### Vital Signs Assessment

#### Overview

The evaluation of the epoch-by-epoch heart rate and breathing rate data collected by the contactless technologies was performed against reference estimates derived from PSG ECG and RIP thorax. The accuracy and reliability of the heart rate and breathing rate estimates were performed at different levels of time resolution to determine use cases in which the contactless technologies can be used. These include accuracy assessment of overnight average estimates; ability to capture overnight trends; variability in vital signs; and accuracy in different sleep stages and at different time resolutions (60-, 10-, and 1-minute intervals) of estimates. All laboratory data analyses were performed over the total recording period of the PSG. At all temporal resolutions of comparisons, only complete or valid pairs of estimates were used.

#### Performance Measures

To assess the accuracy of the vital signs estimates (heart and breathing rates), mean absolute error (MAE) and mean absolute percentage error (MAPE) were used as the primary metrics. MAE and MAPE are used to measure the error in the estimate between the device and the PSG reference vital signs. Bland-Altman metrics such as minimum detectable change (MDC), bias, and limits of agreement (LoA) were also computed to provide an overview of the agreement of the estimates and to allow comparison with the evaluations reported in the literature [45,46]. All the measures are reported with 95% CIs. MDC is the smallest change in the estimate that can be detected by the device that exceeds the measurement error. It is equal to half the agreement width [47],

MDC = (LoA_Upper bound_ – LoA_Lower bound_) / 2 **(1)**

We used intraclass correlation (ICC) with 2-way random effects to measure the reliability of measurement and standardized absolute difference, a directionless Cohen *d*, described by Guruswamy Ravindran et al [35] and Haghayegh et al [47], for measuring the dispersion in the bias. All ICC values (range 0-1) were estimated with α of .05; ranges used for interpretation were as follows: ICC<0.5 (poor), ICC=0.5 to 0.75 (moderate), and ICC>0.75 (good reliability). Apart from the above metrics, the coefficient of determination (*r*^2^, a measure of how close the measured estimates are to the reference, computed using simple linear regression) was also used for the concordance analysis. For estimating the significance of differences between vital signs during different sleep stages, devices, and time courses, we used ANOVA followed by linear mixed effects models with the different groups (devices, sleep stages, and time) as fixed effects (with interactions) and participant as a random effect. MATLAB 2021b was used for all statistical analyses.

#### Acceptable Agreement for Heart Rate

The satisfactory level of agreement between the PSG reference heart rate and the device-determined heart rate was set to an error of 10% or +5 or –5 bpm as recommended by the Association for the Advancement of Medical Instrumentation [42,48].

#### Acceptable Agreement for Breathing Rate

For breathing rate, to the best of our knowledge, no device-specific satisfactory level of agreement is discussed in the literature. Hence, the agreement in breathing rate estimates between human observers is used to set the acceptable level for our evaluation. We set the permissible level of error in breathing rate estimation to be +4 or –4 cpm, as reported by Lim et al [49].

### Breathing Disturbance Estimates

Apart from epoch-by-epoch heart rate and breathing rate, WSA also provides a “snoring” signal, which is a binary variable depicting snore presence detected by the device. In addition, the WSA also provides the summary estimates of snoring duration (WSA snore), breathing disturbance intensity (WSA breathing disorder index [BDI]), and AHI (WSA AHI). The Emfit and Somnofy devices do not generate breathing disturbance measures. Only complete or valid pairs of estimates were used to analyze the summary measures. For WSA BDI and AHI estimates, we explored the relationship between them and the concordance of these WSA estimates to the PSG AHI in the laboratory.

For the WSA snore estimates, we explored the concordance of the all-night snore duration estimated by the PSG snore sensor (ie, PSG snore microphone placed on the side of the neck) and the WSA. The PSG snore sensor data were scored by the Somnomedics Domino software using 30 dB as the snore amplitude threshold and a minimum snore duration of 300 ms. We further explored the differences in the snore intensity as measured by the PSG snore sensor for the epochs determined to contain snore events by the WSA, followed by an exploration of the distribution of the snore events during the different sleep stages.

## Results

### Characteristics of the Study Population

Of the 35 participants, more than half of the participants (n=20, 57%) reported comorbidities, including type 2 diabetes, obesity, arthritis, and hypertension, with concomitant medication. In this study population, the mean heart rate was 62.2 (SD 8.9) bpm (men: n=21, 60%; mean 60.6, SD 9.3 bpm; women: n=14; 64.4, SD 7.8 bpm), and mean breathing rate was 14.7, SD 2.9 cpm (men: 14.6, SD 2.9 cpm; women: 14.7, SD 3.0 cpm), as assessed from the overnight laboratory PSG. The average BMI of the participants was 27.0 (SD 4.8) kg/m^2^, with 17% (n=6, BMI >30) being obese. The mean systolic and diastolic blood pressures measured during screening were 148.7 (SD 16.2) mmHg and 87.0 (SD 9.6) mmHg, respectively. During the clinical PSG, it emerged that 95% (n=33) of the participants in the study had some degree of apnea. Of the participants with apnea, 8 (23%) had severe (AHI>30), 9 (26%) had moderate (AHI=15 to <30), and 16 (46%) had mild apnea (AHI=5 to <15). A total of 10 (29%) participants had PLMI>15, which is similar to the prevalence of periodic limb movement syndrome in community-dwelling older adults [50,51]. Some form of cardiac arrhythmia was found in 51% (n=18) of the participants, with 29% (n=10) of them also having severe or moderate apnea. A detailed description of the population characteristics can be found in the study by Ravindran et al [34].

### Overview of Vital Signs Data

#### Example Case

**Figure 1:**
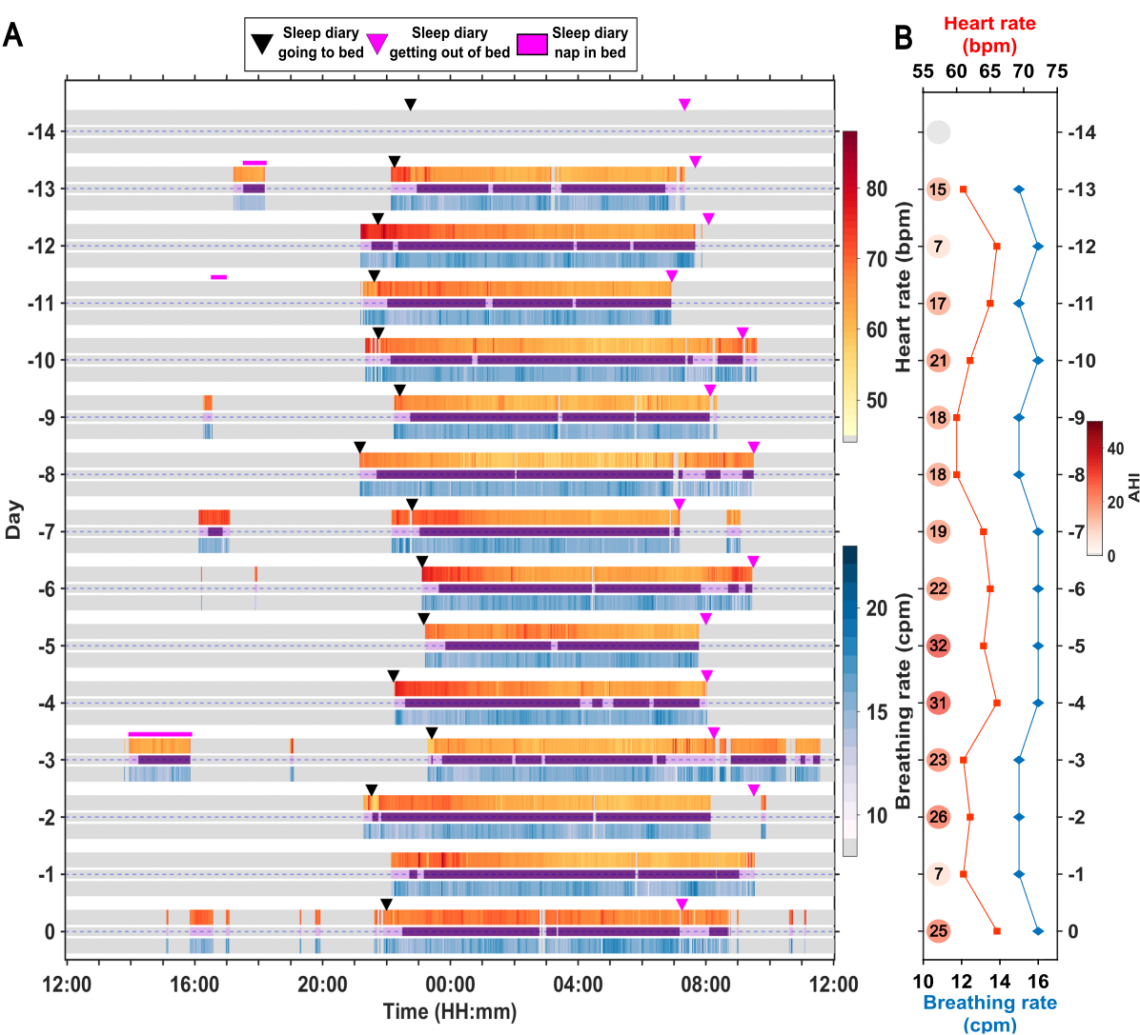
Vital signs from a male participant aged 65 to 70 years collected at home and in the laboratory. (A) Raster plot showing the heart rate (bpm), and breathing rate (cpm) as detected by the Withings Sleep Analyzer along with device-detected sleep (or) wake period and sleep diary information. (B) Estimates of mean heart rate, mean breathing rate, and apnea-hypopnea index (AHI; depicted as circles adjacent to the time courses) were made during the night as determined by the device.

An example of vital sign data collected by WSA for 14 days at home followed by 1 day in the laboratory is shown in Figure 1. The participant had moderate apnea with an AHI of 24.1 events/hour, as determined by the clinical PSG during the laboratory visit (day 0). The raster plot (Figure 1A) shows heart rate, sleep-wake stage, and breathing rate as estimated by the WSA. The vital sign data were available when the participant was in bed at night and during daytime in-bed periods, which were also reported as naps by the participant. The days –14 to –1 depict the data collected at home, while day 0 depicts data collected in the sleep laboratory. The darker, purple-colored regions denote sleep, while the lighter regions denote wake as identified by the WSA. The gray areas represent out-of-bed periods (ie, periods during which the device did not record data). The first day of data from the WSA at home were lost. This participant had irregular nocturnal bed timing, with an average time in bed during the nocturnal period of 10 hours 8 minutes at home. Mean nocturnal heart rate and breathing rate varied across nights. The WSA AHI showed night-to-night variability (Figure 1B), and the WSA AHI during the laboratory visit was 25 events/hour, which was close to the PSG AHI value. The heart rate showed a trend across the nocturnal sleep period, with a higher heart rate at the beginning of the nocturnal sleep period and a lower heart rate just before the end of the sleep period.

**Figure 2:**
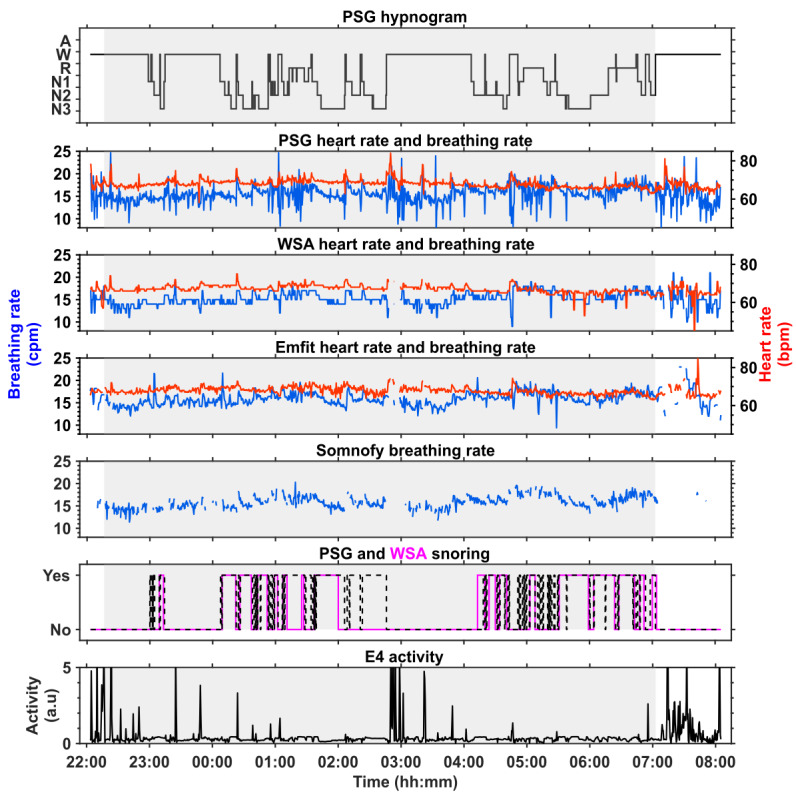
Vital sign data simultaneously collected from 3 contactless technologies and polysomnography (PSG) in the laboratory for the male participant described in Figure 1. The PSG consensus hypnogram (top) is depicted at the top followed by the heart rate (red), and breathing rate (blue) from PSG and contactless devices, and activity data in arbitrary units are from the Empatica E4 device. The gray regions in all the plots correspond to the lights-off period. A: artifact; bpm: beats per minute; cpm: cycles per minute; N1: stage 1 of nonrapid eye movement (non-REM) sleep; N2: stage 2 of non-REM sleep; N3: stage 3 of non-REM sleep; R: REM sleep; W: wake; WSA: Withings Sleep Analyzer.

Figure 2 shows the contactless technology–derived vital sign data collected alongside PSG reference data during the laboratory visit for the participant depicted in Figure 1. The PSG heart rate and breathing rate both show changes as the hypnogram shows transitions between different stages of sleep, with higher variability during wake and REM sleep and lower variability during non-REM sleep. The differences in the data resolution (WSA: 60 seconds; Emfit and Somnofy: 30 seconds) between the contactless technologies can be seen from the plots. The WSA vital signs estimates provided by the device are rounded to the nearest integers, leading to more discretized vital sign data. The trends in the heart rate and breathing rate data recorded by the PSG and the contactless technologies are more similar during the sleep periods compared with the wake periods. The Somnofy had a number of missing estimates of breathing rate during many of the wake epochs determined by the device, and these missing vital sign epochs also coincided with periods of higher activity, as detected by a wrist-worn activity device (Empatica E4). Snoring, as detected by the WSA, followed a pattern that closely followed the snoring signal detected by PSG, with some disagreement in the detection of the snoring event (Figure 2).

#### Summary of Collected Data

In the laboratory study, all 35 PSGs (ground truth or reference data, cohort 1: n=18, 51%; cohort 2: n=17, 49%) were available. The total number of nights of data collected in the laboratory for each of the 3 contactless technologies were 35 for WSA, 16 for Emfit, and 17 for Somnofy. One night of data was lost due to a device malfunction for Emfit. At home, a total of 401 days of data were collected across the 35 participants, with 321 days of data available for WSA (cohort 1: n=10, 56%; cohort 2: n=17, 100%) and 228 days of data available for Emfit (cohort 2: n=17, 100%). At home, for WSA, portions of data from 8 participants were lost in cohort 1 due to deployment errors and Wi-Fi dropouts, with a data loss of 3.3% (11/332 days lost). For Emfit, the data loss was 4.2% (10/238 days lost).

In the laboratory, the range of the heart rate estimated (minimum to maximum) by the contactless technologies was 40 to 90 bpm for WSA and 40 to 135 bpm for Emfit, whereas for breathing rate, the ranges were 8 to 35 cpm for WSA, 6 to 30 cpm for Emfit, and 6 to 30 cpm for Somnofy. The undermattress device–generated vital sign (both heart rate and breathing rate) data for 100% of the in-bed periods. Somnofy bedside radar, by contrast, generated breathing rate data less continuously, resulting in data unavailability at 32.21% (54.41/169.14 hours) of the in-bed period (in-laboratory). Most of these missed breathing rate epochs were found to be in the Somnofy-predicted wake state (the total percentage of breathing rate epochs unavailable per label was follows: wake=34.35/54.41 hours, 62.39%, REM=6.96/54.41 hours, 12.73%; LS=12.19/54.41 hours, 23.1%, and DS=0.91/54.41 hours, 1.78%).

### All-Night Vital Signs

**Figure 3:**
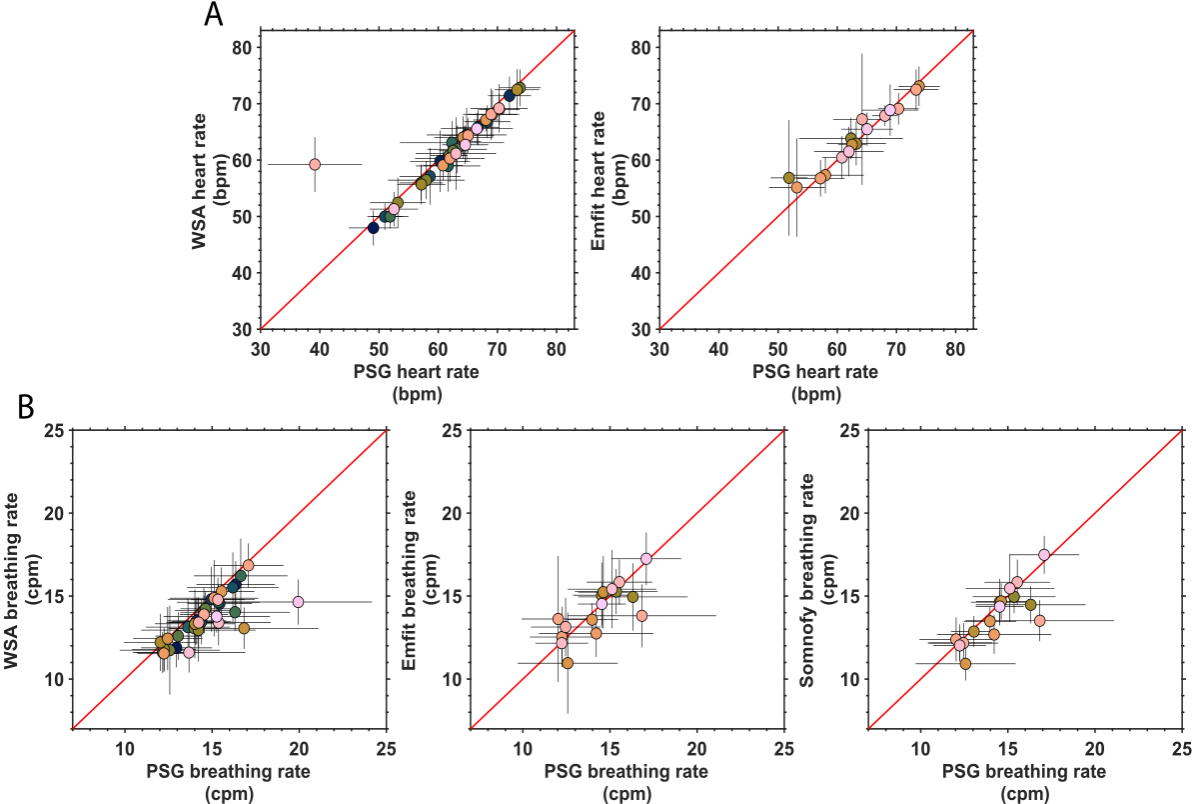
Association between vital signs estimated by 3 contactless devices and estimates from polysomnography (PSG) averaged across the night while sleeping in the sleep laboratory. (A) Heart rate (beats per minute [bpm]); (B) breathing rate (cycles per minute [cpm]). The error bars represent the SD of the estimate within participants. The PSG heart rate and breathing rate are derived from electrocardiogram (ECG) and respiratory inductance plethysmography thorax (RIP thorax), respectively. The number of participants (ie, nights) available for the devices are Withings Sleep Analyzer (WSA; n=34), Emfit (n=16), and Somnofy (n=17).

The concordance between the nightly average heart rate and breathing rate estimates of the contactless technologies against PSG are shown in Figure 3 and Table 1. The WSA (MAPE 3.28%; ICC=0.87) had a lower level of agreement compared with Emfit (MAPE 1.83%; ICC=0.96), and this was due to an outlier participant with severe arrhythmia (see the outlier in Figure 3). When the outlier was removed WSA (WSA*: MAPE 1.87%; ICC=1.0) had an agreement with the PSG that was similar to that of Emfit. The MDC was higher for Emfit (3.25 bpm) compared with WSA* (1.17 bpm), which can be seen from the higher dispersion in the Emfit estimates (Figure 2A). For the breathing rate, Somnofy (MAPE 4.64%; ICC=0.82) had a high agreement, followed by WSA (MAPE 6.29%; ICC=0.78) and Emfit (MAPE 5.46%; ICC=0.76). The MDC follows the agreement results, with Somnofy (1.98 cpm) having a somewhat lower value compared with WSA (2.08 cpm) and Emfit (2.21 cpm).

**Table 1:** All-night average of vital signs and their agreement metrics^a^.

Vital signs			Device, mean (SD)		PSG^b^, mean (SD)		Bias^c^ (SD; 95% CI)		LoA^d^ (lower bound), (95% CI)		LoA (upper bound), (95% CI)		MDC^e^		MAE^f^ (95% CI)		SAD^g^ (95% CI)		MAPE^h^ (95% CI)		ICC^i^ (95% CI)
**Heart rate**																					
	WSA^j^	61.66 (6.51)		62.16 (7.52)		–0.5 (3.63; 1.75 to 0.74)		–7.61 (–9.76 to –5.46)		6.61 (4.46 to 8.75)		7.11		1.69 (2.80 to 0.58)		0.24 (–0.09 to 0.58)		3.28 (0.40 to 6.16)		0.87 (0.75 to 0.93)	
	WSA*^k^	61.73 (6.6)		62.84 (6.46)		–1.11 (0.6; –1.31 to –0.9)		–2.28 (–2.64 to –1.92)		0.07 (–0.29 to 0.43)		1.17		1.15 (1.33 to 0.97)		0.18 (–0.16 to 0.52)		1.87 (1.56 to 2.17)		1 (0.99 to 1)	
	Emfit	63.85 (5.66)		63.42 (6.47)		0.44 (1.66; –0.45 to 1.32)		–2.81 (–4.35 to –1.27)		3.68 (2.14 to 5.22)		3.25		1.08 (1.78 to 0.39)		0.18 (–0.33 to 0.7)		1.83 (0.52 to 3.13)		0.96 (0.90 to 0.99)	
**Breathing rate**																					
	WSA	13.66 (1.38)		14.61 (1.69)		–0.95 (1.06; –1.32 to –0.59)		–3.04 (–3.66 to –2.41)		1.13 (0.5 to 1.75)		2.08		0.97 (1.33 to 0.6)		0.63 (0.3 to 0.97)		6.29 (4.31 to 8.27)		0.76 (0.58 to 0.87)	
	Emfit	14.13 (1.6)		14.35 (1.67)		–0.22 (1.13; –0.82 to 0.38)		–2.43 (–3.48 to –1.38)		1.99 (0.94 to 3.03)		2.21		0.78 (1.22 to 0.34)		0.49 (–0.02 to 1.01)		5.46 (2.55 to 8.36)		0.76 (0.44 to 0.91)	
	Somnofy	13.77 (1.68)		14.28 (1.65)		–0.51 (1.01; –1.02 to 0.01)		–2.48 (–3.38 to –1.58)		1.47 (0.57 to 2.37)		1.98		0.69 (1.14 to 0.24)		0.43 (–0.07 to 0.93)		4.64 (1.79 to 7.5)		0.82 (0.56 to 0.93)	

^a^Metrics of agreement for overall heart rate of the devices against the electrocardiogram (ECG) estimates (included in the PSG). The number of participants contributing to each of these devices was as follows: WSA (n=35), WSA* (n=34), Emfit (n=16), and Somnofy (n=17).

^b^PSG: polysomnography.

^c^Bias is the difference in measurement between the device and PSG ECG.

^d^LoA: limits of agreement.

^e^MDC: minimum detectable changes; smallest detectable change independent of measurement error (half of Bland-Altman agreement width).

^f^MAE: mean absolute error.

^g^SAD: standardized absolute difference; directionless version of Cohen *d*.

^h^MAPE: mean absolute percentage error.

^i^ICC: intraclass correlation coefficient with 2-way random effects; measure of measurement reliability.

^j^WSA: Withings Sleep Analyzer.

^k^WSA* depicts the outlier-removed WSA data.

### Vitals Signs During Different Vigilance States

We investigated the agreement of the vital signs estimated by the contactless technologies to the PSG reference during the different vigilance states of the consensus PSG hypnogram. The distribution of the vital signs for the different vigilance states is provided in Figure S1 in Multimedia Appendix 1. The heart rate and breathing rate estimates of both the PSG and contactless devices were not normally distributed (via the Kolmogorov-Smirnov test). The difference between the mean heart rate estimated by the devices and PSG was <2.5 bpm across all sleep stages. The difference between the mean breathing rate estimated by the devices and the PSG was <1.5 cpm across all sleep stages. Overall, the concordance between the estimates of both heart and breathing rate provided by the contactless technologies and PSG was good across all vigilance states (Figure S2 and Tables S1 and S2 in Multimedia Appendix 1).

### Time Course of Vital Signs During Sleep

**Figure 4:**
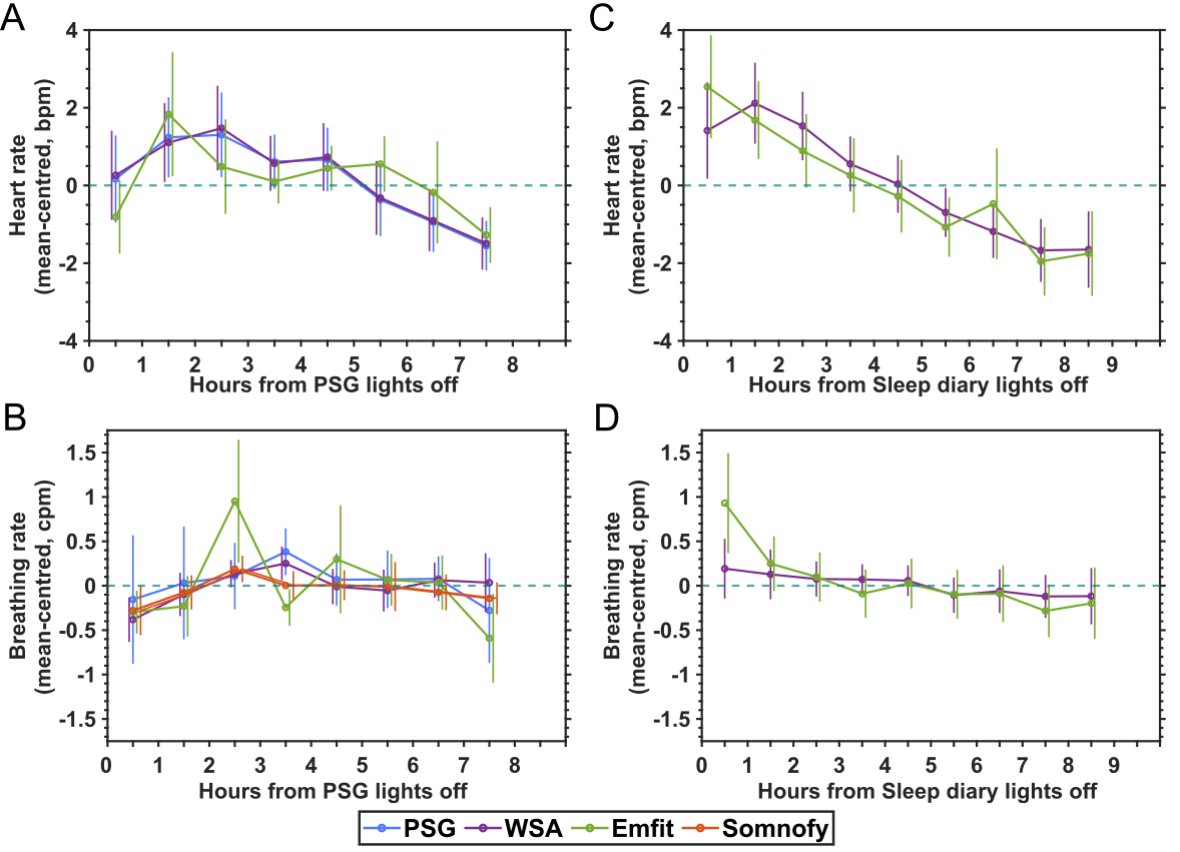
Time course of vital signs during sleep in the laboratory (A and B) and at home (C and D). The number of participants available for each of the devices (measured in nights) in the laboratory (polysomnography [PSG]: n=35, 35 nights; Withings Sleep Analyzer [WSA]: n=34, 34 nights; Emfit: n=16, 16 nights; and Somnofy: n=17, 17 nights) and at home (WSA: n=27, 295 nights; Emfit: n=17, 213 nights). bpm: beats per minute; cpm: cycles per minute.

Figure 4A shows the time course of the heart rate and breathing rate estimated by the contactless devices and PSG over the PSG lights-off period in the laboratory. Figure 4B shows these time courses for the sleep diary–defined lights-off periods recorded at home (Figure 4B). The vital sign data were mean centered and averaged over the epochs during which participants were asleep as detected by the PSG hypnogram for the laboratory data and as detected by the sleep scoring algorithms of the devices for the at-home data. The data are plotted per hour starting from the onset of the lights-off period. The error bars represent the SD of the estimate and are shifted along the x-axis to improve visibility.

Both in the laboratory and at home, the heart rate started close to or above the mean, gradually decreased over the night, and reached the nightly minimum in the second part of the sleep period before a slight increase at the end of the sleep period. The similarity between the contactless technology and PSG heart rate hourly time series in the laboratory was determined using MAPE. The WSA (MAPE 14.42%) closely follows the PSG, while the Emfit (MAPE 144.34%) is less similar.

The breathing rate trends at home were similar to that of the heart rate, starting above the overnight mean and gradually decreasing to a lower value closer to the end of the night. In the laboratory, the breathing rate hourly estimates were fluctuating and did not show any clear trend. Both the WSA (MAPE 129.95%) and Somnofy (MAPE 130.01%) had the highest similarity with the PSG, while the Emfit had the lowest similarity (MAPE 295.76%).

### Effect of Temporal Resolution on the Vital Signs Accuracy

**Figure 5:**
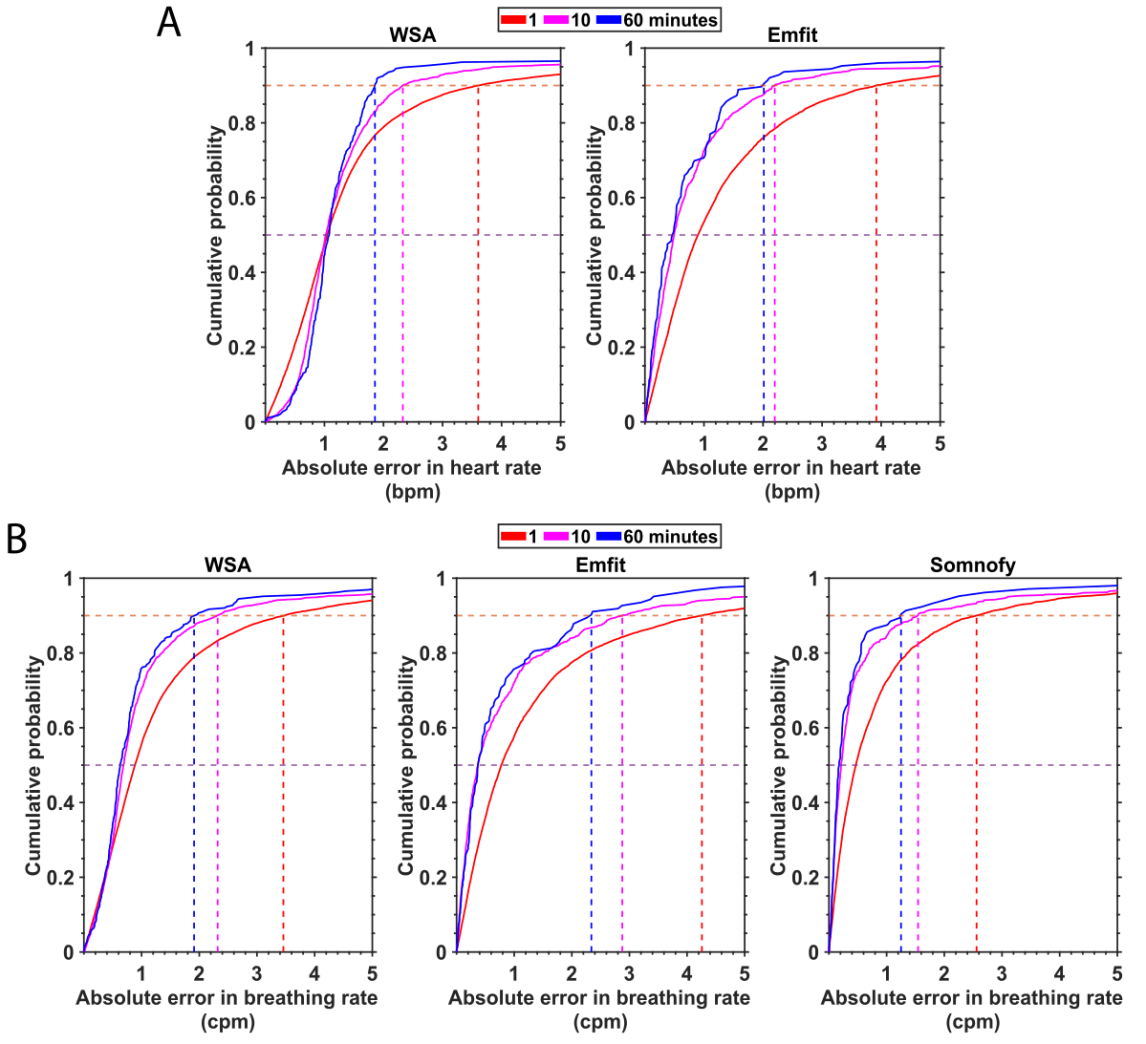
Effect of time window over which vital signs are estimated on device measurement error; (A) heart rate (beats per minute [bpm]) and (B) breathing rate (cycles per minute [cpm]). The cumulative density function of the absolute error is represented for each of the devices for the window lengths 1, 10, and 60 minutes. The median (50th percentile) and the 90th percentile are represented by horizontal lines. PSG: polysomnography; WSA: Withings Sleep Analyzer.

To examine the effect of the length of the time period over which the vital signs are computed, we averaged the heart rate and breathing rate estimates over 60 minutes, 10 minutes, and 1 minute and computed the agreement with the corresponding PSG reference estimates (Tables 2 and 3). We examined the cumulative distribution function (CDF) of the MAE at these resolutions to better characterize the estimation error. The CDFs are depicted in Figure 5, while more detailed scatter plots and associated agreement measures are provided in Figure S3 and Tables S2 and S3 in Multimedia Appendix 1.

For all devices and both heart and breathing rates, the CDFs become steeper with increasing duration of the time window over which these variables are computed. For both heart rate and breathing rate, the agreement (measured by ICC) with the PSG reference estimates increased with decreasing temporal resolution (Figure S3 in Multimedia Appendix 1). On closer inspection of the CDFs, we find that for the heart rate estimates, the error at the 90th percentile is lower for WSA than for Emfit for the 1-minute and 60-minute estimations (overall error<4 bpm). For the Emfit, the median (50th percentile) error of the heart rate estimates became smaller with increasing duration of the time window, but the WSA median error was always close to 1. For the breathing rate, at all 3 resolutions, 50% of the estimates had an error of <1 cpm. When we inspected the 90th percentile error of breathing rate estimates at 1 minute, we found that Somnofy had the lowest error (2.56 cpm), followed by WSA (3.46 cpm) and Emfit (4.26 cpm). This trend was seen for lower resolutions as well. At the 50th percentile, we see the effect of discrete breathing rate output from WSA on the 50th percentile error, where the error of the other 2 devices falls below 0.5, while the WSA error does not. A detailed discussion of the effects of temporal resolution on the agreement between the device and PSG vital sign estimate is provided in Multimedia Appendix 1.

**Table 2:** Effect of temporal resolution on reliability of estimates of heart rate^a^.

Heart rate resolution		Device, mean (SD)	PSG^b^, mean (SD)	Bias^c^ (SD; 95% CI)	LoA^d^ (lower bound), (95% CI)	LoA (upper bound), (95% CI)	MDC^e^	MAE^f^ (95% CI)	SAD^g^ (95% CI)	MAPE^h^ (95% CI)	ICC^i^ (95% CI)
**60 minutes**											
	WSA^j^	61.76 (7.0)	62.09 (8.03)	−0.33 (3.84; −0.78 to 0.12)	−7.86 (−8.63 to −7.1)	7.2 (6.44 to 7.97)	7.53	1.75 (2.15 to 1.35)	0.23 (0.12 to 0.35)	3.46 (2.41 to 4.51)	0.87 (0.84 to 0.9)
	Emfit	64.04 (6.52)	63.58 (6.78)	0.46 (2.85; −0.04 to 0.96)	−5.12 (−5.98 to −4.26)	6.04 (5.18 to 6.9)	5.58	1.16 (1.63 to 0.69)	0.18 (0 to 0.35)	1.95 (1.09 to 2.8)	0.91 (0.87 to 0.93)
**10 minutes**											
	WSA	61.71 (7.18)	62.07 (8.21)	−0.36 (3.94; −0.54 to −0.18)	−8.07 (−8.38 to −7.76)	7.36 (7.05 to 7.66)	7.71	1.83 (1.99 to 1.67)	0.24 (0.19 to 0.28)	3.58 (3.15 to 4)	0.87 (0.86 to 0.88)
	Emfit	64.03 (7.04)	63.57 (7.04)	0.47 (4.01; 0.19 to 0.74)	−7.39 (−7.86 to −6.91)	8.32 (7.84 to 8.79)	7.85	1.4 (1.66 to 1.14)	0.2 (0.13 to 0.27)	2.38 (1.9 to 2.86)	0.84 (0.82 to 0.86)
**1 minute**											
	WSA	61.71 (7.44)	62.08 (8.59)	−0.37 (4.54; −0.43 to −0.3)	−9.27 (−9.38 to −9.16)	8.54 (8.42 to 8.65)	8.9	2.08 (2.14 to 2.02)	0.26 (0.24 to 0.27)	4 (3.85 to 4.14)	0.84 (0.84 to 0.84)
	Emfit	64.02 (7.54)	63.56 (7.44)	0.46 (5.24; 0.35 to 0.58)	−9.8 (−10 to −9.61)	10.73 (10.54 to 10.92)	10.27	2.12 (2.22 to 2.02)	0.28 (0.26 to 0.3)	3.52 (3.33 to 3.7)	0.76 (0.75 to 0.76)

^a^Metrics of agreement for overall heart rate estimates from the devices against PSG electrocardiogram (ECG) estimates (beats per minute) at various temporal resolutions.

^b^PSG: polysomnography.

^c^Bias is the difference in measurement between the device and PSG ECG (device–PSG ECG).

^d^LoA: limits of agreement.

^e^MDC: minimum detectable changes; smallest detectable change independent of measurement error (half of Bland-Altman agreement width).

^f^MAE: mean absolute error.

^g^SAD: standardized absolute difference; a directionless version of Cohen *d*.

^h^MAPE: mean absolute percentage error.

^i^ICC: intraclass correlation coefficient; with two-way random effects (measures of measurement reliability).

^j^WSA: Withings sleep analyzer.

**Table 3:** Effect of temporal resolution on the reliability of estimates of breathing rate^a^.

Breathing rate resolution			Device, mean (SD)		PSG^b^, mean (SD)		Bias^c^ (SD; 95% CI)		LoA^d^ (lower bound), (95% CI)		LoA (upper bound), (95% CI)		MDC^e^		MAE^f^ (95% CI)		SAD^g^ (95% CI)		MAPE^h^ (95% CI)		ICC^i^ (95% CI)
**60 minutes**																					
	WSA^j^	13.67 (1.51)		14.58 (1.87)		−0.91 (1.28; −1.06 to −0.76)		−3.42 (−3.67 to −3.17)		1.6 (1.34 to 1.85)		2.51		1 (1.14 to 0.86)		0.59 (0.47 to 0.71)		6.48 (5.73 to 7.22)		0.72 (0.65 to 0.77)	
	Emfit	14.18 (1.86)		14.33 (1.85)		−0.14 (1.66; −0.44 to 0.15)		−3.39 (−3.9 to −2.89)		3.11 (2.6 to 3.62)		3.25		0.92 (1.16 to 0.67)		0.5 (0.32 to 0.67)		6.35 (4.68 to 8.02)		0.6 (0.47 to 0.7)	
	Somnofy	13.89 (1.65)		14.31 (1.8)		−0.42 (1.19; −0.65 to −0.19)		−2.76 (−3.16 to −2.36)		1.92 (1.52 to 2.31)		2.34		0.55 (0.77 to 0.33)		0.32 (0.13 to 0.51)		3.47 (2.27 to 4.67)		0.76 (0.67 to 0.83)	
**10 minutes**																					
	WSA	13.67 (1.67)		14.6 (2.2)		−0.93 (1.67; −1.01 to −0.86)		−4.21 (−4.34 to −4.08)		2.34 (2.21, 2.47)		3.28		1.14 (1.21 to 1.07)		0.59 (0.54 to 0.63)		7.29 (6.94 to 7.65)		0.63 (0.61 to 0.66)	
	Emfit	14.16 (2.2)		14.35 (2.17)		−0.18 (2.1; −0.33 to −0.04)		−4.31 (−4.56 to −4.06)		3.94 (3.69 to 4.19)		4.12		1.09 (1.22 to 0.96)		0.5 (0.43 to 0.57)		7.42 (6.6 to 8.25)		0.54 (0.48 to 0.58)	
	Somnofy	13.87 (1.77)		14.35 (2.05)		−0.48 (1.49; −0.59 to −0.36)		−3.41 (−3.6 to −3.21)		2.45 (2.26 to 2.65)		2.93		0.68 (0.79 to 0.57)		0.36 (0.28 to 0.43)		4.2 (3.64 to 4.76)		0.69 (0.65 to 0.73)	
**1 minute**																					
	WSA	13.67 (2.04)		14.61 (2.61)		−0.93 (2.32; −0.97 to −0.9)		−5.48 (−5.54 to −5.42)		3.61 (3.56 to 3.67)		4.55		1.51 (1.54 to 1.48)		0.64 (0.63 to 0.66)		9.85 (9.68 to 10.02)		0.51 (0.5 to 0.52)	
	Emfit	14.17 (2.5)		14.35 (2.57)		−0.18 (2.73; −0.24 to −0.12)		−5.54 (−5.65 to −5.44)		5.18 (5.07 to 5.28)		5.36		1.6 (1.65 to 1.56)		0.63 (0.61 to 0.65)		11.07 (10.74 to 11.4)		0.42 (0.4 to 0.44)	
	Somnofy	13.87 (1.91)		14.35 (2.43)		−0.47 (1.99; −0.52 to −0.43)		−4.38 (−4.46 to −4.3)		3.43 (3.35 to 3.51)		3.9		1.06 (1.1 to 1.01)		0.48 (0.46 to 0.51)		6.76 (6.54 to 6.99)		0.58 (0.57 to 0.6)	

^a^Metrics of agreement for overall heart rate estimates from the devices against PSG electrocardiogram (ECG) estimates (beats per minute) at various temporal resolutions.

^b^PSG: polysomnography.

^c^Bias is the difference in measurement between the device and PSG ECG (device–PSG ECG).

^d^LoA: limits of agreement.

^e^MDC: minimum detectable changes; smallest detectable change independent of measurement error (half of Bland-Altman agreement width).

^f^MAE: mean absolute error.

^g^SAD: standardized absolute difference; a directionless version of Cohen *d*.

^h^MAPE: mean absolute percentage error.

^i^ICC: intraclass correlation coefficient; with two-way random effects (measures of measurement reliability).

^j^WSA: Withings sleep analyzer.

### Estimating Breathing Disturbances During Sleep

#### WSA Snore

**Figure 6:**
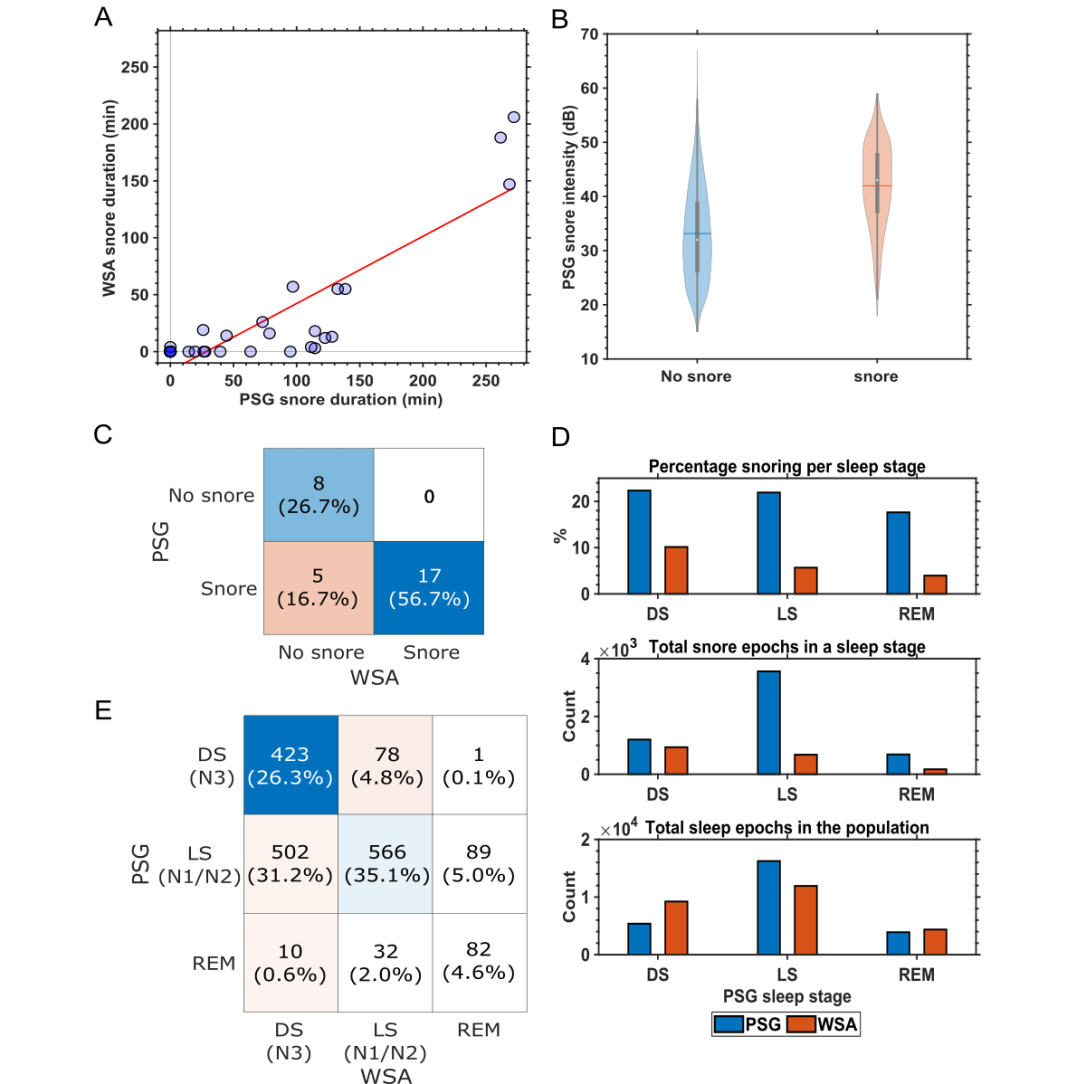
Withings Sleep Analyzer (WSA) snore analysis. (A) Concordance of polysomnography (PSG)-assessed snore duration and WSA snore (n=30). The linear fit is depicted by the red line. (B) Snore intensity as detected by the PSG snore sensor during WSA predicted snore and no snore. (C) Confusion matrix for participants identified as snorers or nonsnorers (n=30). (D) Distribution of snore events across PSG-derived sleep stages for both PSG and WSA. (E) Confusion matrix of epoch-to-epoch (EBE) concordance between PSG and WSA during WSA snore events (total WSA snore epochs: 1611). bpm: beats per minute; cpm: cycles per minute; DS: deep sleep; LS: light sleep; REM: rapid eye movement.

The results of the WSA snore analysis and an example of the overnight time course of the snore data are depicted Figure 6, as well as in Figure S6 in Multimedia Appendix 1. Out of the 35 participants, snore data from both PSG and WSA were available for 30 (86%) participants (PSG snore sensor data were not available for 2 participants, and WSA snore data were unavailable for 3 participants). Of the remaining 30 participants, it was determined from PSG snore sensor data that 8 (27%) participants did not have any form of snoring, whereas 22 (73%) participants snored. The WSA incorrectly determined that 5 (23%) of these 22 participants had no snoring, leading to a moderate performance with a Matthews correlation coefficient of 0.69 (Figure 6C).

PSG determined that the 22 snorers had a nightly snoring duration ranging between 10 and 270 minutes (Figure 6A). The concordance between the snore duration estimates from the WSA and PSG was high (*r*^2^=0.76; *P*<.001; n=30). When the snoring intensity determined by the PSG snore sensor was grouped based on the WSA snore labels (Figure 6B), we found that, on average, the intensity of the PSG-detected snore events that were not detected by WSA was lower than the intensity of the snore events detected by both PSG and WSA, but there was a considerable overlap between the distributions.

Overall, the snore events were underestimated by the WSA compared with PSG. The distribution of the snoring events as determined by the PSG snore sensor showed that snoring was present across all sleep states, with >20% of all non-REM epochs having snore events, while for REM epochs, this was 17.48% (Figure 6D). By contrast, when the distribution of snore events automatically identified by WSA was analyzed, the WSA-determined snore events were high during LS and DS and low during REM (Figure 6E). The corresponding confusion matrix between the PSG consensus sleep stage and Withings sleep stage prediction during WSA-identified snore events is depicted in Figure 6E, which shows that the WSA does not score wake when snore events are detected. WSA also scored more snore epochs as DS, followed by LS and REM sleep.

#### WSA AHI

The WSA summary measures relevant to breathing disorders are the BDI and AHI. WSA BDI data were available for 29 participants in the laboratory and for 24 participants at home (a total of 222 nights available). Both WSA AHI and BDI were available for only 64 nights across 7 participants at home. Upon inspection of potential correlations between the WSA AHI and WSA BDI, we found that the WSA BDI was double the value of WSA AHI (WSA BDI=(2.006×WSA AHI)–0.133; *r*^2^=.999; *P*<.001; n=64; Figure S7 in Multimedia Appendix 1). On the basis of this inference, we used half BDI as the proxy for WSA AHI for the remainder of the analysis.

**Figure 7:**
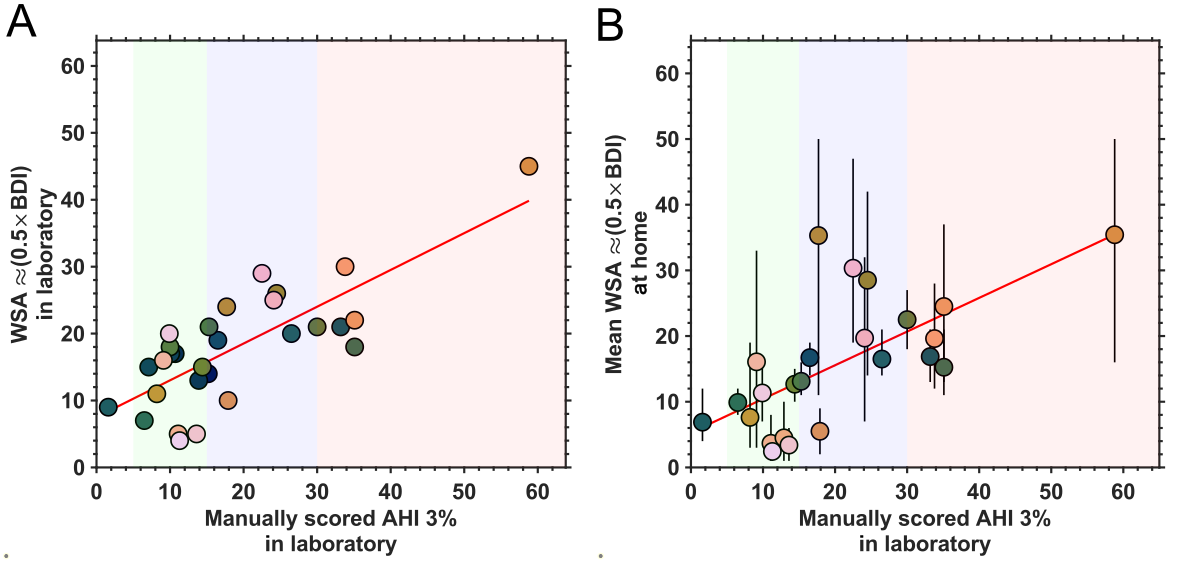
Relationship between polysomnography (PSG)-based apnea-hypopnea index (AHI) and Withings Sleep Analyzer (WSA) breathing disorder index (BDI). (A) In laboratory (n=35) and (B) at home (n=29). The linear fit is depicted by the red line. AHI reference ranges are as follows: 0 to 4 (no apnea), 5 to 14 (mild; shown in green), 15 to 29 (moderate; shown in blue), and ≥30 (severe; shown in red). For the WSA AHI estimates at home, each data point depicts the mean per participant, and the vertical bars depict the minimum and maximum values.

We found a high correlation between the WSA AHI and PSG AHI in the laboratory (*r*^2^=0.59; *P*<.001; n=29; Figure 7A). The MAE was 6.49 (95% CI 4.89-8.10) events per hour. We further explored the relationship between the PSG AHI in the laboratory and the mean WSA AHI at home (Figure 7B). We found that there was a moderate level of correlation between the 2 (*r*^2^=0.44; *P*<.001; n=24). There was also a high level of agreement between the WSA AHI 1 night before the laboratory and both in-laboratory WSA AHI (*r*^2^=0.74; *P*<.001; n=15) and PSG AHI (*r*^2^=0.59; *P*<.001; n=16). We have depicted the WSA AHI (half WSA BDI) for the 24 participants’ data available at home as a heat map showing the night-to-night variability and missing values in Figure S8 in Multimedia Appendix 1.

## Discussion

### Principal Findings

In this study, we provide an evaluation of 3 contactless technologies for monitoring heart rate, breathing rate, and breathing disturbances during sleep in older men and women. Overall, the contactless technologies provided heart rate (WSA and Emfit) and breathing rate estimates (all 3 devices) with acceptable agreement compared with standard reference estimates from PSG ECG and RIP thorax. We also found that these devices can be used for detecting respiratory events, including apnea and snoring, in this population of older men and women with stable comorbidities.

We were able to successfully collect data at home with limited (<5%) data loss. The data loss was primarily due to Wi-Fi dropouts, where the device spontaneously lost connection to the Wi-Fi network. Overall, this demonstrates the ability of these contactless devices to reliably collect continuous vital sign data remotely in the community with little oversight and maintenance.

The devices also captured the time course of vital signs during sleep, in good agreement with the PSG, with relatively small differences in performance between the devices. The heart rate estimate range of both WSA and Emfit was narrower than the Association for the Advancement of Medical Instrumentation–recommended minimum allowable range of 30 to 200 bpm, with WSA having a more limited range compared with Emfit [48]. WSA performed somewhat better than Emfit at capturing heart rate trends, while Somnofy performed the best in terms of breathing rate, followed by WSA and Emfit. The decline of heart rate during the sleep period is in accordance with previous studies, although the upswing in heart rate at the end of the sleep period, which has been observed in younger participants [52], was not very clear in the data collected by any of the methods in this study. This may be because, here, we studied older participants with comorbidities including sleep apnea.

The contactless technologies provided estimates of heart rate and breathing, which, when averaged across the night, were in very good agreement with the PSG estimates. Outliers in both the breathing rate and heart rate agreement plots were found to originate from participants with severe breathing disturbances or significant abnormal cardiac rhythm. The overall agreement between contactless technology–derived estimates and the estimates derived from PSG becomes poorer when the time period over which the estimate is computed becomes shorter. This is not surprising, but it puts limitations on the use cases in which these devices can be applied. Improvement in the estimates from WSA with reducing temporal resolution was limited by the discretized or rounded output of vital signs from the WSA, with 50% of the estimates having a minimum error of 1 bpm.

At 1-minute resolution, the undermattress devices had an acceptable accuracy with an MAE of <2.12 bpm and an MAPE of <5% for heart rate estimates, which is lower than the errors reported in the literature for wearable technologies during daily activities and for many contactless technologies during sleep [24,27]. The breathing rate estimates at 1-minute resolution were acceptable across all 3 devices with an MAE of ≤ 1.6-cpm and an MAPE of >12% and were comparable to other contactless technologies in previous evaluations in young participants [27]. The accuracy of both the vital signs estimates was higher during sleep than in the wake period primarily due to reduced body movements. Somnofy, the best-performing device in estimating breathing rate, was also the best-performing device in our evaluation of sleep stage classification performance [35].

Although in our evaluation of older participants, all the compared contactless devices provided acceptable performance, this performance was poorer than what was previously reported in studies (Emfit and Somnofy) in a younger population. In the evaluation conducted by Ranta et al [32] in a population of 34 participants with a median age of 32 years, the Emfit had an MAE of 1.34 bpm for heart rate and an MAE of 0.59 cpm for breathing rate. In contrast, in the evaluation conducted by Toften et al [31], Somnofy had an MAE of 0.18 cpm in a population of 37 participants with a mean age of 32.6 years. Although the vital sign estimates from WSA have been used in large-scale studies, there is no existing evaluation of the WSA-estimated vital signs in the literature to the best of our knowledge [53,54].

The higher accuracy of Somnofy in estimating breathing rate compared with the undermattress devices can be attributed to the device not estimating breathing rate when the signal quality is affected by body movements. Although this leads to some loss of data, this also leads to better accuracy, highlighting the need for a signal quality index associated with the device-generated vital signs estimates.

Finally, our evaluation revealed that the WSA snore and BDI estimates were accurate, and the performance of the WSA AHI in terms of MAE in our study (MAE 6.49 events per hour; n=29) was better than the results reported by Edouard et al [55] (MAE 9.5 events per hour; N=118; mean age 49.3 years). The variability of WSA AHI across nights, as seen in Figure S8 in Multimedia Appendix 1, highlights the ability of contactless monitoring devices like WSA to capture fluctuations in obstructive sleep apnea and their potential to play a crucial role in understanding changes in daytime function, comorbid conditions, and personalized management.

To the best of our knowledge, WSA snore has not been previously evaluated in other studies. The breathing disturbance detection has been performed using Emfit raw ballistography data in the literature, but these algorithms are not open source or available directly from the manufacturer and hence not used in our analysis [56,57]. The availability of these breathing disturbance estimates, along with the acceptable agreement of the vital sign measures generated by the contactless technologies, demonstrates their immediate potential usefulness in population-wide deployment for home monitoring and care [58].

### Limitations

One of the limitations of the work is that the data synchronization of the PSG reference data and the device data is based on the best alignment of the vital signs data and hypnogram, which is not ideal. Second, the algorithms used by the different devices in deriving the ballistography signal and heart rate and breathing rate information are hidden due to their proprietary nature, and hence the interpretability of several observations made in this study such as the bounded nature of the output vital signs, outliers, and unavailability of data is limited. Due to a lack of detailed documentation on the snore-detection approach used by WSA, the minimum intensity of the snoring that is detected as a snoring event by WSA is unclear. Finally, out of the compared contactless devices, the Somnofy radar does not provide heart rate estimates, which is a limitation.

### Conclusions

With their ability to reliably collect heart rate, breathing rate, and breathing disturbance data longitudinally and at scale, contactless technologies have the potential to be a powerful tool for unintrusive remote vital signs monitoring in community-dwelling older adults and people living with dementia. Applications range from early detection of abnormalities and deterioration of health to monitoring the impact of interventions to improve health (eg, treatment of sleep apnea). This is particularly valuable for patients using prescribed medications for long-term conditions, as it may indicate the need for dosage adjustments or even discontinuation (eg, bradycardia in patients on cholinesterase inhibitors). Together, these applications could improve overall home care. They also allow for investigation into the night-to-night variation in sleep and vital signs and how this variation is associated with health outcomes and daytime function. Such approaches have already shown that night-to-night variation in sleep apnea is associated with uncontrolled hypertension [58] and that night-to-night variation in sleep continuity is associated with day-to-day variation in symptoms in people living with Alzheimer disease [59].

## Appendix

Multimedia Appendix 1 Supplementary tables and figures.

## Abbreviations

AASM: American Academy of Sleep Medicine
AHI: apnea-hypopnea index
AWS: Actiwatch Spectrum
BDI: breathing disorder index
bpm: beats per minute
CDF: cumulative distribution function
cpm: cycles per minute
ECG: electrocardiogram
ICC: intraclass correlation
LoA: limits of agreement
MAE: mean absolute error
MAPE: mean absolute percentage error
MDC: minimum detectable change
PLMI: period limb movement index
PSG: polysomnography
REM: rapid eye movement
RIP thorax: respiratory inductance plethysmography thorax
WSA: Withings Sleep Analyzer

## Data Availability

The data sets generated during and analyzed during this study are available from the coauthor CdM on reasonable request.
